# The New York Dental Association

**Published:** 1858-06

**Authors:** 


### THE NEW YORK DENTAL ASSOCIATION

Held its regular meeting in Bond street, on Wednesday evening, February 17th.—Dr. John Allen in the chair.

The subject of "what is the best material for the base of artificial dentures?" was continued for discussion, and Dr. Roberts resumed his remarks.

On the previous evening he had taken the ground that continuous gum work was the best method of mounting or inserting artificial teeth. He would now take up the different materials, for he did not think that, when discussing a question of this kind, they should take one particular thing as their hobby, and ride it, whether right or wrong,—but should consider each material used as a base for artificial teeth—cheoplastic, gutta percha, vulcanized rubber, gold and platina,—and select as near as they could from these materials, the best. There might be cases where one material would answer better than another, and it was the duty of the dentist to consult the interest of his patient, and use that which would accomplish in the greatest degree of perfection, all that was required. Gutta percha was an auxiliary to the profession that could not be dispensed with in many instances, but it could not be used for permanent work. Silver plates were sometimes used, but no one would consider it best. Gold plates with block teeth he considered a good material, and when well put up, approached very near to natural ones. But he thought the cost of repairing when broken, too great to be generally adapted. He thought single teeth on gold plates, when properly made, capable of doing as much service in eating as any kind of work that was used. But in putting in a set of artificial teeth, they were to restore expression of face, speech, etc., which could not be done by single teeth. Cheoplastic he had never used and thought he never should, as he considered it a base metal, and when worn would discolor. Vulcanite base he had not used, but he thought there were good points about it worthy of consideration. By this they were able to get as perfect an adaptation as with any other kind of material; and this was very important, for if the foundation be correct, they could build upon it with perfect safety. They were less liable to accidents by falling, than most any other kind of work, and many other good points might be mentioned. But when they were done they had a color that did not correspond with the natural gums, and it was not certain but time might effect it, as he thought that most any material of the kind might yield to the influences of the acids of the mouth. Time alone would test it. He considered it the duty of all to use that which would secure the greatest amount of good to the patient, disregarding the time and labor required to produce it. With continuous gum he believed that everything required of a dentist in the insertion of an artificial set of teeth, could be accomplished. He said with this kind of work, teeth could be made to approach so near to nature that they could hardly be distinguished from natural ones. He spoke at some length of the manner in which it restored the speech, enabling persons to sound the letters of the alphabet called dentals. He also spoke of the manner in which they were enabled to restore the expression of the face, and in doing this properly he considered that something more than mechanical skill was required. Here was a demand for artistic dentistry, and he believed the dentist was responsible for a hideous or pleasant expression given to the face of his patient. He described a case he had, where the upper jaw shut inside of the under one, a great distance, which gave a very disagreeable expression to the face, which he remedied by a whole set of continuous gum, articulating them naturally, and altering the whole expression of the face from that of disagreeable to pleasant. He considered that cleanliness could be secured to a greater degree of perfection by this kind of work than any other. He next spoke of the objections that had been brought against continuous gum work, such as the adaptation of the plate, the rigidity, the clattering noise often heard in full sets, the liability to break down, and also the greater weight required in continuous gum work, all of which he considered easily overcome by proper manipulation. He recommended running a three cornered platina wire from the base of the labiel side of the jaw, across the plate, near a point between the first and second bicuspid, which prevented a case from spreading, and increased its strength very much. He considered the weight of no consequence, as the atmospheric pressure was 15 lbs. to the square inch, the mere difference of an ounce or two was not perceptible, and the weight disappeared the moment that the air was exhausted, and the pressure of atmosphere beneath, kept the plate to its place, which nearly extinguished any disagreeable feeling of weight to a patient. The greatest objection that he had been able to perceive, was the manipulations of the furnace, which but comparatively few would ever be able to overcome, for here was the great secret of success in this kind of work, together with a proper material for making it. Some dentists were in the habit of making their own gum and body, and undertaking to put it up without the least instructions; this they called continuous gum work, but it was not. The odor given off from such work, when repaired, would drive every one from the laboratory. To produce a good article, he contended, required experience, and a proper material of body and gum.

Dr. HAWES inquired if, the weightier a piece of work was made, would it not be in proportion more liable to overcome the atmospheric pressure that supported it, and fall.

Dr. ROBERTS agreed that theoretically this was so; but the atmospheric pressure being 15 lbs. to the inch, the difference of one or two ounces in the weight of the piece did not make any practical difference.

Dr. CLARKE inquired if there was any advantage in having a cavity in the plate; or, if where there was a perfect fit, atmospheric pressure was not sufficient without it.

Dr. ROBERTS said he made his air chambers very small, and some of his heaviest cases, where he entirely dispensed with them, he believed to be most successful.

Dr. HAWES said, that in looking over his account books for the last few years, he collected some facts, which he considered always better than theory. Out of 120 sets of single gum teeth, he had to repair one in four; out of seventeen sets of block-work, he had to repair one in three; out of forty-five platina sets, he had four broken, and four others repaired, making in his experience about one in every five and a half sets made of continuous gum work to be repaired. He had always had a good deal of apprehension as to the platina breaking down, but on looking over his books it seemed to come out in favor of the platina. He thought gold work preferable to platina in some instances; but as a general thing, platina was better in regard to beauty of work and cleanliness. He had had but one set of vulcanized rubber, for a lády who required a temporary set, while the other (of continuous gum) should be repairing. On putting in the set of vulcanized rubber, she immediately said, "Why, how comfortable these are—they are better than the others," but on going to the mirror and looking at the color of the gum, she did not like them. This at present seemed the great objection to that kind of work.

Dr. Putnam said it had been remarked by some one since the last meeting, that the discussion of this subject was already narrowed down to that of two materials, viz., platina and the vulcanite compound. It had also been stated that he and Dr. Allen had each a point to gain in placing their materials in as favorable a light as possible before the profession. He disclaimed any such motive on his own part and on the part of Dr. Allen; on the contrary, Dr. Allen's arguments were free and liberal, and designed to instruct. If he needed a witness of his former devotedness and self-sacrifice for the benefit of the profession, he knew of none who could do him better justice than himself (Dr. P.). He had confined himself to the use of continuous gum for several years, before adopting the use of the vulcanite base; and as this discussion continued, should the least importance be given to what he advanced, he simply asked it to be considered as tending as much towards the benefit of those requiring artificial teeth, as that of those having them to make. In its present state of development, he was free to say that much improvement was desirable before the vulcanite material would supercede the continuous gum work in beauty, or more readily attract the eye of the shopping patient; and while he regretted that he could not there present specimens representing the natural gums, he was able to give much encouragement that that desideratum would, ere long, be obtained; and if not with the rubber compound, it would be in a compound which possessed all the virtues that the vulcanite rubber now did. He had learned that two charges were preferred by dentists against the rubber material, viz., that it would soon become soft, and consequently offensive, and second, that it would irritate and heat the gums. As to the first objection, the majority of those present knew to the contrary. As to the second, that it would irritate or heat the gums, he was not prepared to state clearly, that it would not in any instance. He had met with a few cases where it was worn night and day, without the least regard to cleanliness, that the gums appeared inflamed, and in fact were more or less tender to the touch; but on removing the plate after each meal, for a few days, the difficulty was entirely overcome. Of the cleanliness of this material there could be but one opinion, viz., that no secretion, nor the least moisture could find a lodgement between the teeth and the material, and by its perfect adaptation, less foreign matter found its way under, between the plate and gums, than was the case with some other styles. Again, its comparative lightness enabled the operator to extend the features to any desired fullness, without impairing its adhesion to the gums, or requiring the air-chamber. For partial under sets, or one or more teeth upon the upper jaw, it had peculiar advantages, viz: if a clasp was required, the natural teeth would not suffer from the wear, or by a metallic action, as is sometimes the case in the use of metal. A clasp of rubber invariably fitted the tooth so close as to prevent the secretion of food between; and generally less surface of plate was necessary than if metal were used. In most cases the work could be so made as to spring in between the natural teeth, and the fit was so perfect that it was easily kept in place. He did not wish to be understood as discarding entirely the use of platina and continuous gum: in fact he could hardly get along without them, since some of the manufacturers were so tardy in furnishing teeth suitable for vulcanite work; but while he used platina, he set it in a vulcanite base, and thus had a rim which could be relieved if too high, by trimming off, even to the extent of several thicknesses of platina rims, without exposing the continuous body, or otherwise affecting the beauty or strength of the piece. Still another advantage of the continuous gum, when set in rubber, was the increased strength obtained, and its lessened liability to accident. As to the objection of difficulty in repairing, it was not now so monstrous as it once loomed up before his own eyes. To obtain a good impression of a partial piece, he could confidently recommend a preparation of gutta percha and prepared chalk, of equal parts. This softened, in hot water and put into a cup the same as wax, and kept in the mouth from one to two minutes, would yield sufficient, (without change) to give a correct duplicate of any irregularity of a tooth, or teeth, or any overhanging or large crowned teeth, which wax or plaster fails to accomplish.

Dr. Clarke considered that they had devoted a great deal of attention to the best material, without referring back much to the uses of gold. He was sorry they had not some more information on the way of fastening teeth to gold plates. He believed platina was a very good thing, but all his own practice and impressions were strongly in favor of single teeth, set on a gold plate, as being durable, seldom wanting repair, and giving general satisfaction.

Dr. Corell regretted that he was obliged by other engagements to be absent from the greater part of the discussion on both evenings. In relation to continuous gum or rubber, he should acknowledge that he was unprepared to discuss their merits or demerits. He supposed he was rather an

"old fogy," but he had adhered to the gold plate with tenacity. His opinion was, that ordinary gum teeth, if properly put up, on a gold plate, would give as good satisfaction as any that could be put up. They were not so nice in appearance as continuous gum, but he felt confidence in the one, because he had tested it; and he should say that he did not hear as much complaint from the gold plate, with block or single teeth, as he did from continuous gum work. He had no doubt, however, that they were making constant improvements—and perhaps continuous gum, or perhaps vulcanite rubber, would ere long become the thing chiefly, if not altogether used.

Dr. FRANKLIN remarked that there seemed to be some question in regard to the strength of every kind of work spoken of. Now what constituted the strength of a gum tooth? It was the platina pin. If the backing was too strong, under a given amount of pressure, the tooth gave way; and if the tooth was too strong then the backing bent. They had, in continuous gum work, the same pin and a thicker tooth to start with, and if there was any strength at all in the silicious compound out of which the gum was composed, that was an additional strength to any set singly or on blocks. Dr. F. described his method of putting up continuous gum work; and after a very extensive experience, he considered it, when properly put up, the strongest kind of work. In the appearance it gave the face, he could tell a set of single teeth as far as he could see them. The face looked superanuated, dried up, gone. The continuous gum, on the contrary, brought back the expression of youth. As to the objection of weight, he had found the heaviest pieces made, gave great satisfaction, and he believed a little weight, more or less, made no practical difference.

The committee reported the "filling of teeth" as the subject for next meeting, when the association adjourned to Wednesday evening, February 24th.

